# Psychosocial and Physical Rehabilitation of Burn Survivors: A large multicentre cluster randomised controlled trial from Pakistan

**DOI:** 10.1192/j.eurpsy.2023.1919

**Published:** 2023-07-19

**Authors:** N. Chaudhry, M. panagioti, A. B. Khoso, T. Kiran, A. Blakemore, K. Lovell, M. M. Bashir, Z. U. Haq, M. Hashmi, Z. H. Suhag, H. Brooks, E. McIntosh, N. Soomro, S. Falder, L. Kynge, J. Miah, O. Eylem, S. Edwards, R. Memon, S. J. Pocock, Z. Zadeh, I. B. Chaudhry, N. Husain

**Affiliations:** 1 Pakistan Institute of Living and Learning, Karachi, Pakistan; 2 University of Manchester, Manchester, United Kingdom; 3 King Edward Medical University, Lahore; 4 Khyber Medical University, Peshawar; 5 Ziauddin University, Karachi, Pakistan; 6 University of Glasgow, Glasgow, United Kingdom; 7 Sindh Institute of Physical Medicine and Rehabilitation, Karachi, Pakistan; 8 The Interburns, Wales; 9 Manchester Global Foundation, Manchester; 10 University College London; 11 London School of Hygiene and Tropical Medicine, London, United Kingdom

## Abstract

**Introduction:**

Globally, burns are responsible for around 11 million injuries and 180 000 burn-related deaths yearly. Unfortunately, 9 of 10 burn injuries and deaths happen in low-and-middle-income countries (LMICs) such as Pakistan. One in three people admitted to hospitals with burn injuries die within three weeks, and survivors face serious lifelong physical, emotional and psychosocial problems. This may result in anxiety, depression, post-traumatic stress disorder, increased mortality and social disintegration. This study aims to evaluate if implementation of a culturally adapted multidisciplinary rehabilitation programme for burn survivors is clinically and cost-effective, sustainable and scalable across Pakistan.

**Objectives:**

- To understand lived experiences of burn survivors, families, and other stakeholders including the experience of care and impact of burns To work together with key stakeholders (such as burn survivors, family members) to adapt a culturally appropriate affordable burn rehabilitation programme
- To undertake social media campaigns to promote burn prevention and risk assessment at communities, workplaces/industries/households; improve first aid; and address burn related stigma
- To work with policy makers/parliamentarians to develop national guidelines for burns care and prevention in Pakistan

**Methods:**

There are 6 work-packages (WPs). WP1 is to co-adapt a culturally appropriate burn care and rehabilitation programme. WP2 will develop and implement national burn registry on WHO’s initiative. WP3 is a cluster randomised controlled trial to determine clinical and cost-effectiveness in Pakistan. WP4 will evaluate social media campaigns for burn prevention and reduce stigma. WP5 involves working with key-stakeholders for burns-related care and policy and WP6 offers sustainable capacity and capability for burns treatment and rehabilitation.

**Results:**

A clinical and cost-effective burn care quality and rehabilitation programme may have a huge potential to save lives and contribute health and socio-economic benefits for patients, families, and the healthcare system in Pakistan. The nation-wide implementation and involvement of burn centres across all provinces offer an excellent opportunity to overcome the problem of burn care access experienced in LMICs.

**Conclusions:**

To date, burns prevention, care and rehabilitation have not received sufficient attention in policy initiatives in Pakistan and other LMICs. This study is an excellent opportunity to evaluate culturally adapted burn care and rehabilitation programmes that can be implemented across LMICs. We will disseminate our findings widely, using a variety of approaches, supported by our stakeholder and patient advisory groups.

**Disclosure of Interest:**

None Declared

